# Supporting teachers in a war zone: a mixed methods study of a brief intervention in the context of ongoing war

**DOI:** 10.1186/s13031-026-00775-4

**Published:** 2026-02-28

**Authors:** Gerlinde C. Harb, Majed Qishawi, Steffi Gebus, Jon-Håkon Schultz

**Affiliations:** 1 UiT the Arctic University of Norway, Tromsø, Norway; 2 Norwegian Refugee Council, Gaza, Palestine

**Keywords:** War, Teachers, Self-care, Intervention, Peer support, Palestine, Gaza, Trauma, Emergency, Crisis, Education in emergencies

## Abstract

**Background:**

In the ongoing war in Gaza, teachers face a dual burden as caregivers supporting traumatized children and as community members exposed to the same violence and trauma. Teachers are therefore at high risk for burnout and supporting teachers’ well-being is critical during war. In the context of extreme stress and crisis, supporting those who deliver psychosocial support to children is essential. This paper presents the development, implementation and piloting of an intervention to improve teachers’ psychological well-being self-care during war.

**Methods:**

A bottom-up approach was used to assess the needs and difficulties of Gazan teachers using “listening sessions,” and a group intervention was co-developed in collaboration with those living and working in the war zone. A mixed-methods design was employed for the pilot study, utilizing quantitative data from a pre- and post-assessment tool completed by teachers in Gaza (*n* = 33) and in the West Bank (*n* = 24), alongside qualitative data from listening sessions with teachers, steering committee meetings, and field notes.

**Results:**

Listening sessions revealed that Gazan teachers felt overwhelmed by intense and ongoing traumatic events and loss alongside significant ongoing external stressors. Based on these findings, a 3-session intervention focusing on self-care and peer support was developed and piloted. Quantitative data showed significant pre-post improvements in well-being (*t*(48) = 7.48), coping skills (*t*(48) = 7.13), and sense of meaning *t*(49) = 3.56), while qualitative findings highlighted increased cognitive flexibility and focus through social support, new awareness and insights, and coping strategies.

**Conclusions:**

This brief and feasible support intervention for teachers showed significant very positive effects on their subjective sense of well-being and coping in the midst of war stressors. It is expected that the teachers’ improved coping during wartime will also increase their ability to positively interact with the stressed and traumatized children they support in educational settings.

## Background

In contexts of extreme stress and crisis, supporting those who provide psychosocial support to children is imperative, underscoring the need for targeted interventions aimed at enhancing teachers’ capacity for self-care. This paper describes the development of an intervention for teachers in the context of the ongoing war in Gaza, as well as its implementation and piloting. The current project was started in the summer of 2024, approximately 300 days into Israel’s ongoing war on the Gaza strip that followed the Oct 7th attacks. By the summer of 2024, casualty estimates - likely a vast underestimation [1] - showed that over 35,000 Palestinians had been killed (including over 14,500 children), over 85,000 people injured, 1.7 of 2.2 million residents displaced from their homes and almost 40,000 Palestinian children had lost at least one parent [2]. The dire humanitarian situation also included the destruction of 60–80% of buildings, halted education and destroyed school infrastructure, and extreme levels of food insecurity [2]. Thus, it was clear that the life of all children and adults was impacted on all levels by the ongoing bombing, destruction and deprivation.

These impacts must be understood within the context of nearly two decades of military siege, occupation and major military escalations [3, 4]. Thus, the civilian population of Palestine was routinely exposed to potentially traumatic events as well as deprivation, displacement and disruption in schooling/employment, leaving a mark on their mental health and well-being [5–8]. The Norwegian Refugee Council (NRC) is a humanitarian organization that promotes education as a fundamental human right and an integral part of humanitarian emergency responses [9]. Recognizing that children in emergency contexts must manage their psychological stress responses to the crisis in order meaningfully engage in education, the NRC together with UiT The Arctic University of Norway (UiT) developed a psychosocial support program for refugee children, the Better Learning Programme (BLP; [10–12] This stepped psychosocial support intervention was implemented in most schools across Gaza and the occupied West Bank, delivered by teachers and school counselors within the school setting between 2012 and 2023. After the beginning of the war on Gaza in 2023, the increased psychosocial needs of the displaced, stressed and acutely traumatized vulnerable civilian population, including children, was quickly evident [13]. The NRC began to gradually re-introduce learning environments for children in the midst of the war in February of 2024, in the form of aptly termed “temporary learning spaces”. Due to the systematic destruction of learning spaces and school buildings and the erasure of educational institutions in Gaza [14], these educational safe spaces were temporarily housed in tents in various displacement camps rather than resembling typical school buildings. In the face of this war on education, creating safe spaces for learning was essential. Simultaneously, the occupied West Bank experienced a sharp increase in violence against civilians and displacement during this time [15] prompting humanitarian emergency interventions in refugee camps. Teachers previously trained in teaching psychosocial coping skills with the BLP began providing organized support and structure to groups of children in both Gaza’s displacement camps and West Bank refugee camps.

Teachers providing educational activities and psychosocial support for children during ongoing violence, deprivation and trauma experience a dual burden as both caregivers/teachers and survivors/community members. Especially during this war on the educational infrastructure, teachers take on the critical role of “frontline workers” in education, as teachers are often an essential part of the support system for children in traumatic environments (e.g. [16]. Teachers in crises have been shown at risk for burnout, secondary traumatization as well as mental health deterioration [17]. Moreover, teachers are simultaneously responsible for supporting children’s emotional stability while also managing their own responses to the same stressors and potentially traumatic events (as documented recently in Ukraine [18]. Life during war carries with it a heavy mental health burden due to continuous trauma exposure that has been documented in many contexts [19–23]. When teachers are affected by their own acute stress responses, their capacity to support children’s needs is likely impacted, consistent with evidence indicating that teacher well-being is an important determinant of child outcomes [24, 25]. Thus, supporting teachers’ mental health and well-being during wartime becomes an important focus of the emergency intervention and their psychological coping should obtain increased research attention [17].

While many programs have been developed to increase self-care for teachers (for recent review, see [5–8] or self-care for front-line workers (e.g. [26, 27], these programs cannot be readily adapted to settings of daily bombardment, multiple displacements, and limited privacy. The current program aims to fill the notable gap in the literature on self-care programs for scalable, rapid-response psychologically focused self-care interventions designed for educators in active armed conflict. The urgency and complexity of teachers’ needs in Gaza underscores the importance of developing a “self-care package” that is both culturally and contextually grounded and straightforward, time-efficient and impactful. Examination of existing self-care interventions showed that most were either resource-intensive, aimed at people in comparatively advantaged social positions, or dependent on stable environments, features not compatible with an active war setting.

A collaborative, bottom-up approach to developing this intervention was a fitting method for designing an intervention in this context. This type of co-design has been recommended in global mental health as a means of ensuring cultural and contextual validity [28]. The current paper describes the development process of the Better Learning Programme for Teachers (BLP-T) and its rapid implementation and piloting. The research objectives for the current report were formulated as follows:

1. Describe and document the support needs of the population and the procedure for the development of this intervention.
2. Evaluate quantitatively whether the participants’ perceived coping and well-being increased from pre- to post-intervention in an open trial design.
3. Explore qualitative data on the implementation and impact of the intervention.

## Methods

### Procedures

The current protocol was designed according to the Guidelines for Research Ethics in the Social Sciences and Humanities established by the Norwegian National Research Ethics Committee (2024) and approved by the NRC. A formal plan for managing personal qualitative data through recordings was approved by the Norwegian Agency for Shared Services in Education and Research (Sikt). Additionally, the study received approval from the Research Ethics Committee of the Faculty of Humanities, Social Sciences, and Education (HSL) at UiT. Further, an NRC technical steering committee composed of psychosocial support leaders at global, regional and local levels and three of the authors (GCH, MQ, JHS) met bi-weekly to oversee the study’s implementation.

The collaboration between NRC and UiT was to support NRC in their delivery of educational support for children in emergency situations. The collaboration included identifying and prioritizing support of unmet needs and led to the design and piloting of BLP-T. In the war context, research was defined as a secondary activity, with the primary goals of supporting activity in line with NRC’s work, that is, designing procedures for measuring, evaluation and learning (MEL) to be integrated in NRC’s standard procedure for the use of BLP-T. In this regard, the research process became an integrated part of the work, led by UiT, without taking the focus away from NRC’s main priority of supporting children during war. NRC and UiT went through a joint process of exploring and understanding possible risks and define safety measures. The risk assessments were shared with and approved by the ethics committee of UiT and remained an active document to be discussed and revised throughout the collaboration.

Conducting research in active armed conflict requires adapting standard procedures to ensure non-traceability to not place participants at risk. International guidance for research in emergencies – including the Council for International Organizations of Medical Sciences International Ethical Guidelines and the Declaration of Helsinki – recognizes that oral consent may be used when written records increase vulnerability. Though written consent was not ethically appropriate, all teacher participants provided verbal informed consent after discussion of the project with group leaders, but no identifying information was recorded.

#### Positionality

The researchers (GHS, JHS) who engaged directly with teachers, group leaders and teacher trainers in Gaza were identified as researchers and psychologists, which may have introduced power dynamics. However, this was mitigated by the collaborative atmosphere of all meetings, and the desire to defer to local experts and participants was emphasized. The NRC employee researcher who was coordinating the project in Gaza (MQ) had close involvement in all pilot groups in Gaza. To minimize social desirability bias, all outcome assessment was in writing and collected by the NRC Monitoring, Evaluation and Learning team. Reflexive practices included sharing preliminary findings with group leaders and conducting supervision and fidelity meetings with researchers (GHS, JHS). The researchers (GCH, JHS and MQ), who participated in the development of BLP-T (i.e. listening sessions, supervision and handbook development), were not involved in the initial phase of the qualitative data analysis (independently conducted by SG). The preliminary findings were subsequently discussed and further developed through joint reflection with the full research team.

#### Listening sessions

To assess the needs of Gazan teachers in the temporary learning spaces, two 1.5 h virtual “listening sessions” examined what self-care means in the context of war with six teachers, an NRC teacher trainer (MQ), and a translator. The first session covered their current difficulties, while the second session also examined teachers’ reactions to the first listening session. The sessions followed a discussion format guided by two authors (GCH, JHS). Discussion questions included: “What are your daily challenges? What is going well?”, “How are you taking care of yourself?” and “What are the obstacles to taking care of yourself?” The outcomes of the sessions are described in the results section and lead to the development of the BLP-T’s structure and content.

#### Pilot groups

After the development of the session outlines, piloting took place in two general locations over the course of 10 months (between November 2024 and September 2025): the Gaza Strip and the West Bank. The first five groups were conducted in different locations in the Gaza strip, with three group leaders with significant experience training teachers in the BLP facilitated the groups between November 2024 and February 2025. Each group consisted of six to eight teacher participants who had on average 2.5 (SD = 1.37) years of prior BLP teaching experience and were currently providing psychosocial support and educational activities for children in displacement camps.

Five groups were conducted in the West Bank between July 2025 and September 2025. The groups were conducted in locations in which NRC recently implemented psychosocial crisis interventions for children. Locations of increased violence and conflict were chosen: Yatta, Dora/Al Tabaq town, Al Fawar camp, Beit Omar and Balata camp/Nablus. While different from the war setting of the Gaza groups, teachers in the West Bank groups also experienced ongoing military threat, losses, traumatic experiences and daily danger to their lives and families.

#### Supervision/fidelity

While traditional fidelity evaluations were impossible due to the situation, structured weekly supervision and fidelity meetings were held (virtually or in person) with the group leaders and at least one of the authors. These meetings reviewed the content and process of the conducted groups, helped to trouble-shoot challenges, and supported skillful application of BLP-T principles.

### Research design

This study employed a two-phased mixed-methods approach based on Creswell’s mixed methods evaluation design [29], combining quantitative data from pre- and post-assessments with qualitative data from recordings and fieldnotes of listening sessions, discussion meetings with authors and NRC employees, as well as supervision meetings. The first phase followed an exploratory sequential design, using qualitative data from listening sessions, discussion sessions and meetings to guide the development of BLP-T in Gaza and to develop the quantitative assessment tool. The second (explanatory design) phase first utilized this assessment tool to assess changes in teachers’ self-reported coping strategies, emotional states and well-being. Subsequently, qualitative data from recordings and fieldnotes gathered during additional steering committee and supervision meetings was used to understand the assessed changes and explore the experiences and perspectives of the group leaders.

### Measures and participants

#### Pilot group participants

Between the two locations, 57 teacher participants were enrolled in the program and completed the pre-intervention assessment. All 57 participants completed the three BLP-T sessions, however, due to circumstances beyond our control in Gaza, the post-assessments of seven participants were lost. Thus, 50 participants completed both pre- and post-assessments. In Gaza, the participants enrolled in the five groups consisted of a total of 33 participants with an average age of 28.67 (SD = 2.68) and were evenly split in terms of gender (i.e., 50% female). All participants reported that they had been displaced from their homes an average of 4.8 times (SD = 2.7) at the time of enrollment. The 24 participants in the West Bank groups with average age of 30.42 years (SD = 7.80) were predominantly female (91.7%).

#### Quantitative data collection

***Measures***: Limited quantitative assessment was possible due to the context of this project during an active war, therefore a concise assessment measure was created by two psychologists (GCH and JHS) rather than using extensive pre-existing scales. The pre/post assessment of coping and well-being was developed for this research and therefore no reliability data are available, limiting generalizability of the current results. The assessment was stream-lined to include basic demographics (gender, age, location) and for Gaza groups, the length of time participants had been teachers and how often they had been displaced. The assessment of self-care included 5 questions rated on a 4-point Likert scale, ranging from “0 = Never” to “3 = Always”. The following aspects of self-care were assessed by single items: physical/bodily (“I am able to take care of my body’s needs (food, drink, sleep, medical care etc.).”), mental/thoughts (“I am able to calm myself and manage my worry.”), emotional/feelings (“I can cope with my feelings.”), interpersonal/social (“I am able to get support from my community and/or family.”) and purpose/meaning (“I feel that I can make a positive change through my work.”). One open-ended question asked participants to identify current feelings, and two questions assessed subjective overall well-being (“In general, how would you rate your overall mental well-being over the last week?”) and coping (“In general, how would you rate your overall coping over the last week?”) on a 10 point Likert Scale (0 = very poor to 10 = very good).

***Analysis***: All statistical analyses were performed using the SPSS 29.0.2 statistical analysis software and included descriptive statistics to describe sample characteristics as well as pre- and post- functioning. The main quantitative research question was assessed with within subject t-tests for pre-post changes and follow-up analysis using independent samples t-tests and one-way ANOVAs; Cohen’s *d* effect sizes were also calculated.

#### Qualitative data collection

***Data***: Qualitative data consisted of fieldnotes from (1) steering committee meetings (not audio recorded), alongside audio recordings and fieldnotes from (2) discussion meetings between authors and NRC employees, (3) two listening sessions and (4) supervision meetings with six group leaders. In total, approximately 80 pages of fieldnotes and transcriptions were generated from 41 online meetings.

***Analysis***: The qualitative analysis was conducted by one author (SG) who was not involved in the program development and meetings that generated the qualitative data to enhance analytical distance. A deductive, top-down approach was applied, using categories derived from the quantitative assessments guiding the coding framework: Teacher need for support, peer support for teachers and improved well-being among teachers. The initial codebook was developed based on these categories and refined iteratively during the coding process. All written materials were coded line-by-line in NVivo [30]. An initial set of 179 codes was reviewed, refined, and consolidated into 49 codes, which were then developed into overarching themes. Themes were subsequently discussed and cross-checked with other authors for credibility and coherence. The coding process and development of themes were clearly documented to ensure transparency, and a consistent approach was applied to enhance dependability and replicability.

## Results

The results section first presents an overview of the stressors, needs and barriers for self-care in Gaza’s BLP teachers, followed by results of the quantitative analyses, and concluding with the qualitative findings.

### Teacher need for support

Teachers and group leaders provided descriptions of their current struggles with fear, loss, daily responsibilities and trauma, recognizing that in Gaza “everyone is injured” and “in need of healing.” Table 1 lists the barriers to self-care that teachers reported, including external and internal stressors of living the crisis of ongoing war. As can be seen, internal stressors are clearly interconnected with, and often reactions to the realities of the external stressors. However, despite these circumstances and their own emotional struggles, teachers returned to work: “The Gaza teachers are still standing: All infrastructure, schools and houses – most of it is gone. The only thing we have still standing are the teachers. And they’re volunteering for teaching.”

**Table 1:** Current stressors and barriers to practicing self-care

External stressors and barriers: conflict and crisis context	Internal stressors and barriers: emotional strain and overwhelm
Unsafe environments: K*eeping self/family safe* Unstable conditions: V*olatility*,* need to frequently flee to new displacement areas* Physical limitations: B*eing displaced*,* lack of space*,* restriction of movement* Life threat and basic survival needs: L*ack of water*,* food security*,* hygiene and poor-quality shelters*,* witnessing destruction* Logistics: D*ifficulty reaching work*,* complex life in displacement* Economic strain: L*ack of salary*,* worsening economy*	Triggers of loss and trauma: A*ctivation of personal traumatic and loss memories* Emotional contagion: A*bsorbing children’s distress and fears* Exposure to suffering: W*itnessing others in need* Balancing responsibilities: S*truggling to manage family responsibilities alongside work* Constant worry: F*ear for family’s safety and well-being* Sleep disturbances: *Nightmares and difficulties to relax and sleep* Hopelessness: F*eelings of despair*,* questioning purpose of life and work*

One teacher shared, “life feels hopeless and there is no reason to feel hopeful,” while another highlighted the connection between their distress and the students’: “When we feel stress, we have many of the same reactions as our students. Stress influences the way we think and act. In this war, we are not the best versions of ourselves.” In temporary learning spaces they encountered children with high levels of dysregulation, describing them as “aggressive, impulsive and disorganized.” At the same time, teachers were worried about their families at home, as one teacher explained, “During a working day when I am teaching at the temporary learning spaces, there might be an attack close by – and I keep wondering if any of my family was killed during that attack.”

BLP-T group leaders (who shared the reality of living with similar challenges) also reported experiencing high levels of stress in their own lives and those of the participating teachers. They described the current situation as unprecedented, stating, “It has never been this bad in my lifetime.” They emphasized, “Everyone has lost everything, so we no longer dwell on it. What we do talk about is the loss of life – many teachers have lost a son, a mother, someone close.” One group leader shared a personal account: “I was so afraid for my daughter. I couldn’t protect her. I asked myself: Is this the moment we are going to die?”

Despite the overwhelming circumstances, the team underscored the importance of self-care for the teachers, particularly during times of unpredictability and evacuation warnings. The pilot group leaders highlighted that there is a critical need for a collective space where teachers can listen to each other’s struggles and share their challenges and pressures. One group leader emphasized:

- Our teachers need support. We see that they are now starting to show serious signs of being worn out… We need them to endure as teachers and to provide support for themselves and the students.

### Development of the BLP-T sessions

The outcome of the listening sessions was a better understanding of the teachers’ current stressed state and their needs for increased self-care. The second listening session also provided feedback about teachers’ experiences during the first listening session. Teachers reported a positive effect on their well-being and that despite initial fear or reluctance to discuss their struggles with the team, the shared conversation about their stressors and current coping provided emotional relief. Teachers described positive aspects of the collective conversations to include an avenue for emotional expression, hearing other perspectives, creating safety, and feeling understood in their shared suffering.

The BLP-T approach was subsequently co-developed in close collaboration with the NRC education teams, group leaders, and teachers on the ground, ensuring that the program was shaped by the realities and priorities of those living and working in extreme stress. Local staff repeatedly emphasized that the intervention “is made for them in their situation,” and that its coherence ultimately “is held together by the context and the teachers,” rather than by external advisors. Ongoing feedback guided contextual adaptations throughout piloting, with teachers’ own experiences directly informing what was developed: “This is directly from our experience,” as one NRC colleague underlined. This contributed to an emerging emphasis on *realistic* rather than idealized self-care—an approach grounded in what is possible within an ongoing crisis. As one team member put it, “Now self-care is not an option – it is the highest priority,” not as an added burden but as a means to “keep going and do something good for ourselves and others.” This grounded, experience-based understanding of what is feasible in protracted crisis directly informed the structure and focus of BLP-T, ensuring that the intervention responded to local needs and could truly support teachers within their current conditions.

The theoretical frameworks that subsequently gave structure to the development of the current intervention included models of trauma-informed care, stress and coping models, resilience, and social support theories. While many aspects of self-care are discussed in the literature (e.g. physical, mental, emotional, interpersonal, practical, spiritual), a useful summary definition of self-care provided by Martinez and colleagues [31] was used: “the ability to care for oneself through awareness, self-control, and self-reliance in order to achieve, maintain, or promote optimal health and well-being.” Many of the targets of intervention of other self-care packages were outside of the scope of intervention in this crisis context, therefore the current brief intervention for teachers in the Gaza war was focused on psychological self-care, i.e. coping with thoughts and feelings to improve well-being. Rather than taking a theory-driven approach to creating this intervention, theoretical frameworks informed the continued refinement, implementation, and interpretation of the intervention.

#### Better learning program-teachers (BLP-T)

The handbook for this intervention [32] was refined based on a series of discussions and while adjustments were made during piloting, the structure and content of the intervention remained largely unchanged. The intervention consists of three group sessions of approximately 60–90 min each, conducted one week apart. Group leaders follow a structured handbook containing a session structure, discussion questions and homework assignments. Homework assignments for teachers consist of tracking their stress levels and committing to coping skill practice.

To accommodate the fact that the participants were living in an environment of high to extreme stress, we opted for a de-focused approach over the three sessions, starting with discussing questions about their students (e.g. “Have you noticed any results from using the BLP-techniques with students?” and “How are the coping strategies helping the students?”). Through process of describing their students’ stress reactions and coping strategies, teachers themselves were gradually sensitized towards observing active stress reactions as well as coping strategies. Teachers were then asked about their stress reactions in the teacher’s role, and their ways of coping. Finally, the focus turned to themselves as people, as wives or husbands, as parents and family members.

After the first and second sessions, teachers are also given structured homework assignments (monitoring stress levels and daily practice of coping strategies) to be discussed in the following sessions. The BLP-T is aimed at helping teachers in emergency situations to (1) increase their awareness of their own stress reactions and (2) increase resilient coping (that is, coping with stressful circumstances using an adaptive, flexible problem-solving approach; [33, 34] by practicing a number of different coping strategies. Since BLP teachers already have basic knowledge about traumatic stress, calming exercises and strategies for emotion regulation, the goal is to expand their own coping repertoire by actively using the knowledge and experience from their BLP teaching for themselves.

### Impact of sessions: quantitative results

#### Changes in coping/well-being

**Fig. 1:**
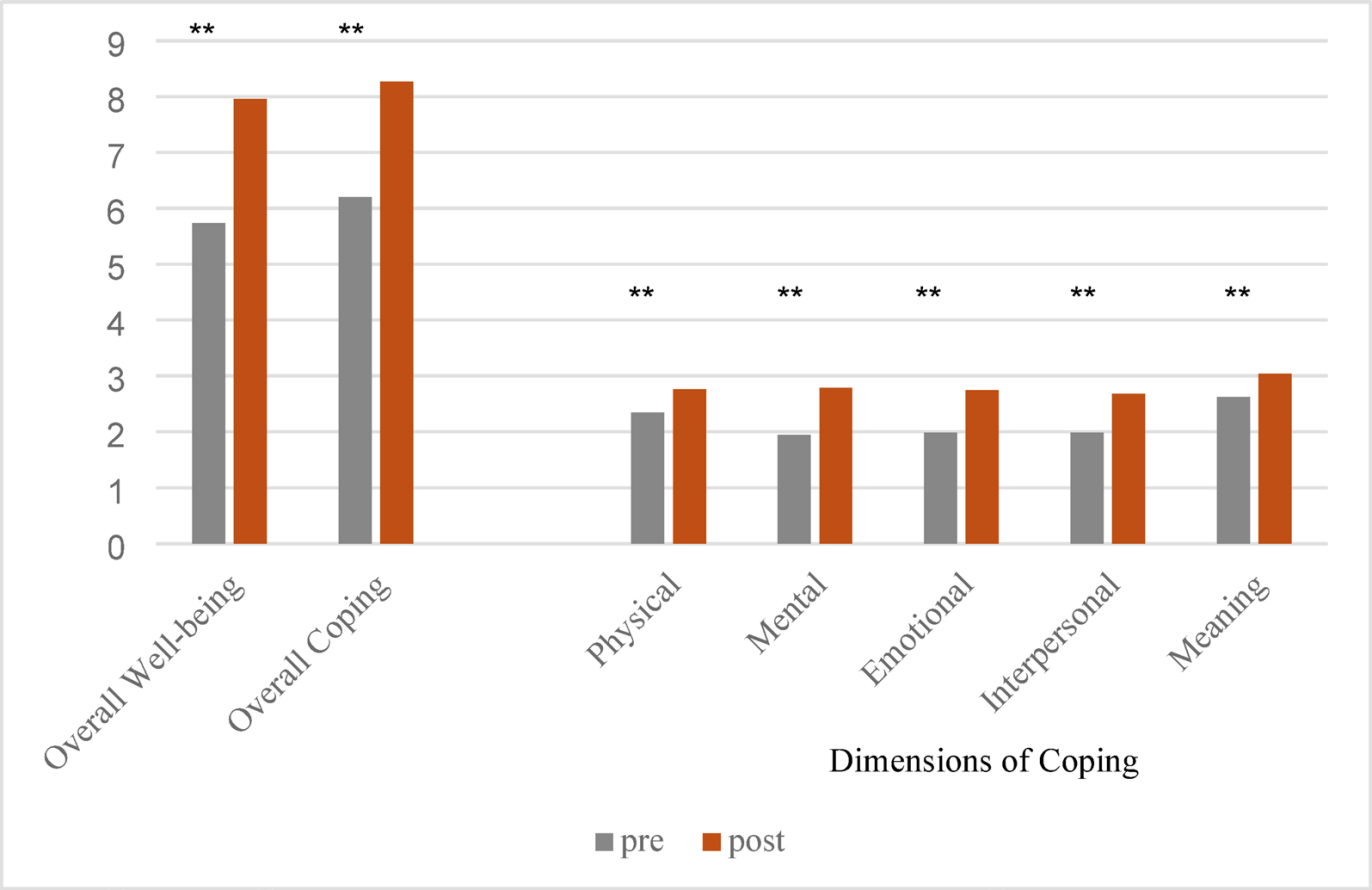
Average pre-post changes for overall well-being and coping, and dimensions of coping (*N* = 50). ** *p*<.001

Pre and post means and standard deviations are presented in Table 2. Both subjective well-being (*t*(48) = 7.48, *d* = 1.07) and coping (*t*(48) = 7.13, *d* = 1.02) increased significantly from pre- to post-assessment (*p*<.001). All dimensions of coping demonstrated a significant (*p*<.001) increase post intervention (physical coping: *t*(49) = 4.23, *d*=0.60; mental coping: *t*(49) = 6.08, *d*=0.86; emotional coping: *t*(49) = 5.73, *d*=0.81; interpersonal coping: *t*(49) = 5.08, *d*=0.72; meaning: *t*(49) = 3.56, *d*=0.50). Pre–post changes reflected large within-subject effects for well-being and coping, with moderate to large effects across coping domains. Figure 1 shows the pre-post changes.

**Table 2:** Pre-post changes in overall well-being and coping and dimensions of coping

Wellbeing^a^	Pre	Mean	SD
		5.73	1.735
	Post	7.96	1.398
**Coping** ^a^	Pre	6.20	1.915
	Post	8.27	1.238
**Dimensions of Coping** ^b^			
**Physical**	Pre	2.34	0.798
	Post	2.76	0.687
**Mental**	Pre	1.94	0.843
	Post	2.78	0.545
**Emotional**	Pre	1.98	0.869
	Post	2.74	0.600
**Interpersonal**	Pre	1.98	0.915
	Post	2.68	0.653
**Meaning/purpose**	Pre	2.62	0.878
	Post	3.04	0.570

^a^10-point scale; ^b^4-point scale

Of the 49 valid pre-post assessments, 78% indicated improvement in coping, with 37% reporting significant 3–6 point improvement on the 10-point scale, while 18.4% reported no change and 4% a slight decrease in coping. Regarding well-being, 77% of the 48 valid participants reported better well-being, with 44% reporting large (3–7 points) improvements, 10.4% no improvement, and 12.5% a 1-point decrease in coping at post-test.

#### Does location matter?

Subjective sense of well-being and overall coping increased significantly more in Gaza than in the West Bank (*t*(46) = 2.19, *p*=.033, *t*(45) = 3.02, *p*=.004, respectively). The mean increase in well-being was 2.81 (*SD* = 1.63) for Gaza and 1.55 (*SD* = 2.34) for the West Bank, while the mean increase for coping for Gaza was 2.81 (*SD* = 2.15) and 1.22 (*SD* = 1.51) for the West Bank. Changes in the dimensions of coping were not significantly different between the two locations (physical: *t*(48)= 0.43, *p*=.67, mental: *t*(48)= 0.82, *p*=.42, emotional: *t*(48)= 0.83, *p*=.41, interpersonal: *t*(48)= 0.35, *p*=.73, and meaning: *t*(48)= 0.31, *p*=.76).

**Fig. 2:**
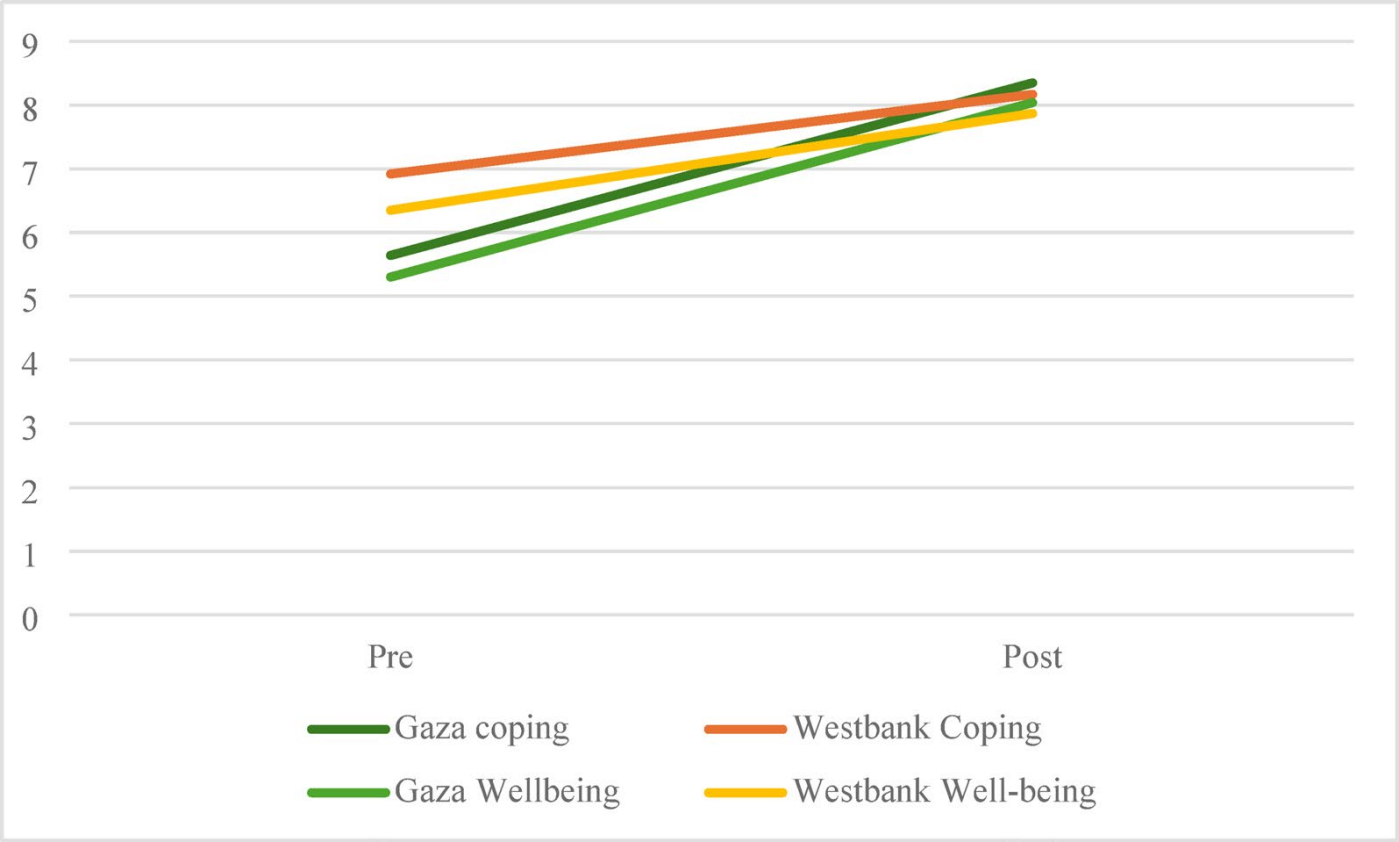
Interaction between location and improvements in coping and well-being

One-way ANOVAs comparing pre-post change scores demonstrated a significant location x time interaction for both coping (*F*(1,47) = 8.74, *p*=.005) and well-being (*F*(1,46) = 4.81, *p*=.033), with a significantly larger gain for Gaza groups for both variables. The between-group comparisons of change scores indicated a moderate effect favoring Gaza for well-being (*d* = 0.62) and a large effect for coping (*d* = 0.85). This interaction effect is exhibited in Fig. 2. Gaza groups were significantly lower in coping and well-being at pre-treatment (*t*(55) = 2.61, *p=*.006 and *t*(54) = 2.39, *p=*.01, respectively), reaching similar levels as the West Bank groups at post-treatment (*t*(47) =0.48, *p=*.32, *t*(47) =0.42, *p=*.34).

### Impact of sessions: qualitative results

The analysis of the qualitative material supports the quantitative results in that is also exemplified improved well-being among teachers after participating in BLP-T. Teachers described the positive impact of BLP strategies on their students, noting that children became calmer, more open about their feelings, and better able to cope with stressors. Over time, teachers began to reflect on their own well-being, demonstrating greater self-awareness and increased flexibility in managing stress, regulating emotions, and recovering from challenges more effectively. Teachers reported feeling more patient, both as educators and parents, and described a renewed sense of purpose in their work. As one teacher shared, “I used to think that life was hopeless and that there was no reason to feel hopeful, but through the sessions, I realized the importance of my existence and the purpose of my work.” By balancing their attention between their students’ needs and their own, teachers felt more energized, motivated, and equipped to navigate the shared stressors of their environment. Teachers expressed a clear interest and willingness to actively address their struggles and utilize their coping strategies as means for improving their self-care.

A review of the 49 codes in the NVivo output resulted in the development of overarching themes to (1) understand the benefits of the intervention and (2) describe group leader perspectives on positive facilitator skills and session challenges.

1. **Understanding the Benefit of the Intervention**.

**Fig. 3:**
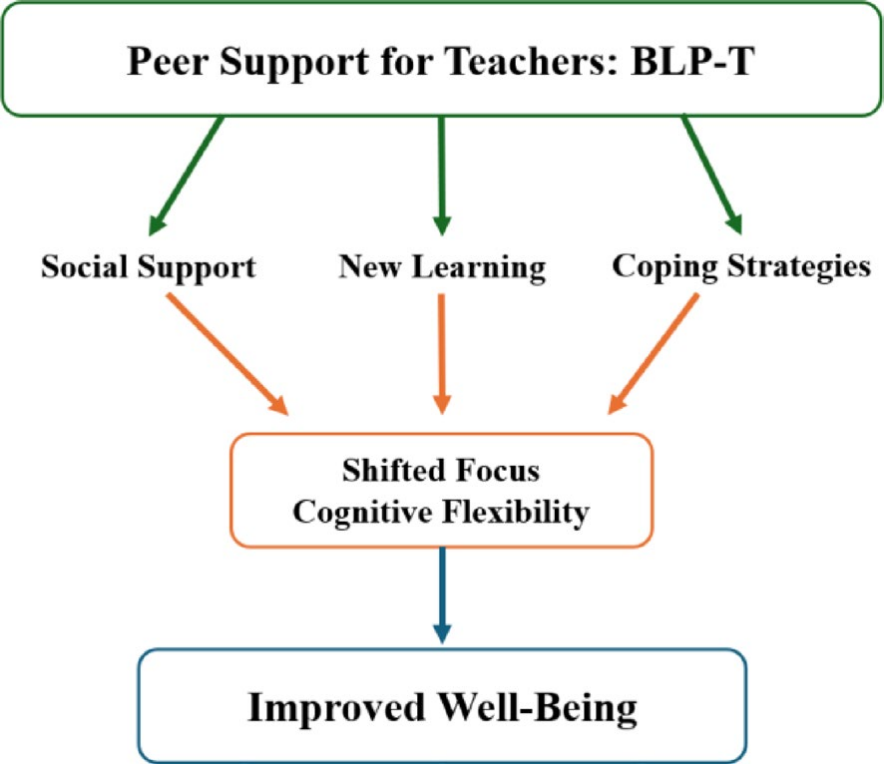
Explanatory themes for teacher improvement

Figure 3 presents a visual overview of the three themes that emerged from the explanatory qualitative analyses.

#### Coping strategies

The sessions encouraged participants to redirect their attention from stressors to practical coping techniques (e.g. breathing, *Safe Place* imagery, muscle relaxation, concentration and self-talk) when recognizing distress. Group leaders played a key role in maintaining this focus on coping, gently steering discussions away from deeply distressing topics while encouraging participants to share and learn from each other’s coping mechanisms. Teachers reported using a variety of coping strategies in addition to those emphasized in BLP such as walking, praying, reading the Quran, and spending time with loved ones to manage their stress. Teachers learned new strategies from each other and learned to use their existing coping strategies in novel ways, such as practicing breathing exercises in calming outdoor locations, like one teacher who shared, “Whenever I’m stressed, I stand on my land and do the breathing exercises, and I manage to switch off and no longer focus on the problems.” Others incorporated *Safe Place* techniques into their nightly routines, with many female participants using the strategy after completing their daily work, often late at night, to calm themselves and restore balance. These techniques not only resulted in more varied and flexible coping but also helped them create routines that supported their well-being in both personal and professional contexts. As one participant shared, “By coming back to myself, I can restore my calmness and routine and continue my life more easily.”

#### Social support

The peer support sessions provided a judgment-free space where participants could openly share their struggles and experiences without fear of shame or judgment. Many teachers shared relief in discovering that their colleagues faced similar challenges, which fostered a sense of solidarity and mutual support. One teacher highlighted: “This is the first time I share my problems with colleagues and was sort of relieved to learn that they face the same struggles”. The sessions helped normalize emotions and reactions, creating a sense of empathy and connection among participants. In addition, teachers found benefit in getting ideas from each other through brainstorming as a group, helping them “find solutions together,” and supporting each other in implementing new solutions that worked for others in the group. Within the safety of the group, teachers were able to shift attention from the stressors and challenges to active solutions.

The social bonds formed during the sessions extended beyond the program, with teachers continuing to meet, support one another, and even organize group activities like shared meals and outings. WhatsApp groups were created to maintain contact and provide ongoing encouragement. As one teacher noted, “It gives me some positive feelings when talking to my friends. There are always some sort of problems that we sort out together. That helps me to feel a little better.” By fostering peer support, the sessions not only helped participants address their own stress but also empowered them to support others in their communities, creating a ripple effect of care and emotional resilience.

#### New learning and insights

The last theme found in the qualitative materials was the new learning and insights they developed over the course of the three sessions. Teachers learned to better understand their own stress responses and reactions, as well as those of their students. Teachers expressed relief and clarity upon learning more about the symptoms of trauma and stress reactions, with one participant sharing, “Now I can understand why this is happening to me in terms of feelings, behavior, and even thoughts.” The sessions emphasized recognizing physical and emotional stress responses, such as muscle tension or intense fear or anger, and directed teachers towards tools like breathing exercises to help manage these reactions. This newfound self-awareness not only enhanced teachers’ self-regulation but also deepened their empathy and understanding of their students’ behaviors, reframing them as trauma responses. As one teacher reflected, “These concepts let me discover new positive things inside myself,” while also fostering a greater sense of connection with the children they support.

2. **Group Leader Perspectives & Challenges**.

Group leaders were described to play a central role in BLP-T, creating a safe and trusted space for participants, ensuring that discussions remain supportive and focused on coping strategies rather than going too deeply into trauma memories and stressful details. They model healthy behaviors, facilitate open communication, and guide participants toward practical solutions. Leaders also use humor and lightheartedness to ease the emotional weight of the sessions, helping participants feel more comfortable and engaged. Despite the challenges of managing emotions and difficult stories, group leaders found the sessions personally rewarding, with one stating, “I feel very proud of myself. This is having a huge impact on the teachers.”

Effective group leaders demonstrated strong facilitation skills, the ability to observe group dynamics, and the capacity to manage emotional discussions. While leaders do not need to be psychologists, they must be well-prepared to handle sensitive topics and guide participants toward coping strategies, such as breathing exercises and *Safe Place* techniques. Training and supervision are critical to building these skills, as even experienced education staff may lack experience in leading adult groups. Leaders must also remain adaptable, tailoring their approach to the unique needs of each group and maintaining a balance between structure and flexibility. As one facilitator noted, “We prepare ourselves very well to handle the sessions to have a good impact on teachers and to see happiness on their faces”.

Finally, group leaders reported several challenges in implementing BLP-T sessions, deeply influenced by the unstable and crisis-driven context as well as cultural and logistical barriers that shaped the experiences of both participants and facilitators. The instability of the situation, marked by regular attacks, arrests, and displacement, created significant barriers to planning and implementing sessions. In addition, participating teachers sometimes struggled to find time and privacy to practice techniques between sessions, especially in overcrowded living conditions or with the constant presence of drones. The overwhelming responsibilities at home and work also often left participants with limited capacity to prioritize their own well-being, despite recognizing its importance. The emotional weight of the sessions was also challenging for the group leaders themselves, as they navigated their own stress while supporting participants. As one leader noted, “You have the feeling of control, but then you just don’t. There is no safe place inside Palestine.”

Cultural aspects also posed challenges: traditional norms in Palestinian society often discourage open discussions about emotions, especially fear and vulnerability [35]. Further, men tended to be more hesitant to share their reactions or difficulties (a possible expression of “scripts of restrictive emotionality” [36]), while women were more open to discussing their emotions. Group leaders had to carefully manage the participants’ gendered and cultural expectations while maintaining focus on the practice of positive coping and increasing emotional resilience.

## Discussion

During the ongoing war and its concurrent attack on education in Gaza, the teachers supporting children in temporary learning spaces in Gazan displacement camps needed support for their own well-being to maintain their teaching capacity. The BLP-T was developed during the current war on the Gaza strip using a bottom-up approach and successfully piloted as an intervention for teachers in emergency situations in Gaza and the West Bank. “Listening sessions” provided the teachers’ perspective of their own needs during war, were an effective starting point for developing the brief group intervention and revealed a willingness and readiness to talk about coping strategies. The idea of “co-design” of interventions together with stakeholders and beneficiaries has been used in global health policy in order to define problems and to develop new solutions in different settings, developing interventions that are grounded in the experience of those who they are trying to help [28]. Defining problems and solutions with the teachers in Gaza allowed us to bridge the gap between research and practice, making the intervention applicable to teachers’ needs and feasible for the context. The resulting program is an intervention that is simple in structure (it can be completed within 3–4 weeks, focuses on “realistic” self-care) and scalable to help many teachers in an emergency (as a group intervention, it serves 6–8 teachers at a time).

The 3-session group program is aimed at building teachers’ psychological resources to cope more flexibly with war-time stressors. The peer support group format of this intervention was an effective approach to harness the power of social connectedness, normalization and shared problem-solving in this setting of collective traumatization. Quantitative evaluation of this uncontrolled outcome study demonstrated significant effects of the intervention, as both overall well-being and coping (including physical, mental, emotional, social and purpose) increased from pre- to post-assessment. Teacher’s well-being improved significantly both in Gaza and the West Bank, with a larger improvement seen in the intense acute war setting in Gaza. The analysis of the qualitative material complements the quantitative results, highlighting that teachers felt more equipped to recognize their emotional reactions and to cope with their ongoing stressors after participating in BLP-T.

The qualitative analyses allowed for an examination of possible reasons behind the success of the intervention by evaluating the feedback from teachers and group leaders. Peer support provided teachers with coping strategies, social support, and new insights, which facilitated a shift in focus toward acknowledging their own stress reactions and fostered more flexible and resilient coping. Individuals who experience trauma and/or prolonged extreme stress often exhibit restricted cognitive flexibility, that is a reduced capacity of adjusting to changes in situations, shifting from one idea to another or choosing varied strategies for different problems [37, 38]. Stress interferes with executive functions and can lead to rigidity in perception, attention allocation and thinking. The cognitive inflexibility characteristic of the traumatized brain can lead to a difficulty in coping that may not be due to lack of coping skills but due to difficulty in the flexible application of skills. Teachers themselves noted an acute awareness that they were less able to use their usual skills during this crisis. The teachers furthermore reported a benefit from an increased awareness of their own repertoire of coping skills, observing others’ coping and receiving suggestions for a variety of strategies they could experiment with, which likely fostered cognitive flexibility. Increased cognitive flexibility might also be protective against the negative effect of future stress in this population as it has been shown to be positively related to active coping [37]. Furthermore, a recent study of Palestinian mental health professionals in the West Bank also underscores the importance of active coping in well-being and preventing mental health difficulties [39].

Using the BLP with children provides teachers with tools to help children calm themselves and to manage their stress to enable them to focus on learning in the midst of the chaos of war. The BLP-T empowers and encourages teachers to use the same strategies to improve their own coping and psychological and emotional state. The defocused approach of BLP-T appeared to have two positive effects. First, it allowed teachers who are normally focused on teaching children coping tools, to turn these techniques towards themselves. This encourages active problem-focused coping, which has been shown to be related to decreased stress levels among teachers [40]. The teachers are encouraged to “make the switch” by first being sensitized through the de-focused approach and then switching the focus towards their teacher role and ultimately themselves as a person. Second, the process of focusing on their teacher role is emphasized in this program to also increase teachers’ sense of purpose and fulfillment as teachers. Sharifian and colleagues (2023) showed that Syrian teachers who felt more fulfillment through teaching during ongoing conflict exhibited more well-being and resilience in their teaching roles. Similarly, Ukrainian teachers recently reported both their professional sense of teaching and helping children as well as joy they derived from teaching were protective factors, helping them cope with war-time stressors [41]. In the Palestinian context, recent research in Gaza showed how war-related obstacles to learning and psychological distress were also connected to narratives of personal strength and resilience in Gazan undergraduate students, resulting in “a collective ethos of endurance and a national spirit of educational resistance, a refusal to let war completely disrupt academic progress” [42]. An increased sense of purpose as teachers should be an important target of self-care and well-being for teachers during war, just as a focus on meaning is part of mental health interventions for frontline aid workers [43]. It is important to note that although this project aimed to support teachers in coping more effectively with wartime stressors, Shwaikh cautions against placing the burden of resilience on survivors of war without simultaneously calling for an end to the oppression and violence inflicted upon them [44].

Societal norms and gendered expectations might have complicated the process of fostering open communication and peer support during this intervention [35, 36]. The BLP-T requires openness about discussing stress, difficulties and emotions, however, cultural expectations may require more “restrictive” emotionality, which prescribes which emotions may be appropriate to be shown to others and when [36]. Norms around emotional expression are further complicated for Palestinians living under military occupation and in a crisis when mental health care may not be viewed as a priority. The current findings highlight the complexities of studying and delivering psychosocial support in a setting marked by ongoing conflict and societal norms that may discourage open discussions about mental health and emotional well-being. These challenges underscore the need for ongoing supervision, training, and support for group leaders to ensure the sustainability and effectiveness of the program in such a complex and volatile environment.

Despite the program’s positive impact, several limitations of this project must be noted and point toward directions for future research. First, the design of this open trial limits the causal conclusions to be drawn from the results, as intervention effects could be attributable, for example, to regression to the mean or other nonspecific variables. Furthermore, the short-term outcome assessment did not allow for an evaluation of the durability of the improvements in coping. Second, the war context required the use of feasible brief assessment instruments, forgoing obvious advantages of using validated and more comprehensive measures of various psychosocial variables. The self-report nature of the assessments may also have resulted in outcomes influenced by social desirability and shared method variance. Future studies may include structured interviews of both group leaders and teacher participants in addition to evaluating the outcomes of children supported and taught by the teachers in the BLP-T groups. This will allow for an evaluation of the practical significance to examine whether better teacher well-being translates to down-stream improvement in students’ emotional regulation and well-being in conflict settings. Third, data were obtained in a specific context and cultural environment, and replication in other emergency and war contexts is needed for generalizability. Such use in novel contexts will require thoughtful adaptations to different cultural contexts. Furthermore, the logistics and specifics of each emergency and humanitarian situation may require further adaptation of the program specifics to increase the likelihood of successful scalability.

## Conclusions

The current study demonstrates that supporting teachers’ well-being during an active war is both feasible and meaningful. The Better Learning Programme for Teachers (BLP-T) was designed as a brief, contextually grounded intervention developed in response to the acute needs of teachers living the realities of trauma, displacement and loss. The pilot intervention demonstrated significant pre-post improvements in teachers’ subjective sense of coping, well-being, and sense of meaning. It is noteworthy that teachers’ subjective sense of well-being and coping increased although their external situation remained replete with factors challenging teachers’ physical and mental well-being. The benefits of peer support and the focus on flexible application of coping strategies emerged as important variables in qualitative analyses. By fostering self-awareness, broadened coping reservoir, and improved cognitive flexibility, this program not only enhanced teachers’ professional skills but also appears to have supported their personal growth and strengthened their capacity to navigate stressors. Supporting teachers in active war is not only important to strengthen their personal well-being, but also an investment into the quality of psychosocial support for children and the rebuilding of an educational structure damaged by war. Future research should extend these findings in other conflict settings with varied and validated measures, control groups and longer-term outcome assessment for both teachers and children. It is, however, paramount that future research in war settings should balance the need for establishing a rigorous evidence base about individuals’ psychological functioning during wartime with the sensitivity and protection of those individuals’ needs.

## Data Availability

The data analyzed during the current study are available from the corresponding author on reasonable request.

## Abbreviations

BLP: Better learning programme
BLP-T: Better learning programme for teachers
NRC: Norwegian refugee council
UiT: UiT the arctic university of norway
